# Prediction of T Cell Epitopes from *Leishmania major* Potentially Excreted/Secreted Proteins Inducing Granzyme B Production

**DOI:** 10.1371/journal.pone.0147076

**Published:** 2016-01-15

**Authors:** Ikbel Naouar, Thouraya Boussoffara, Mehdi Chenik, Sami Gritli, Melika Ben Ahmed, Nabil Belhadj Hmida, Narges Bahi-Jaber, Rafika Bardi, Yousr Gorgi, Afif Ben Salah, Hechmi Louzir

**Affiliations:** 1 Laboratory of Transmission, Control, and Immunobiology of Infections-LR11IPT02, Pasteur Institute of Tunis, Tunis, Tunisia; 2 University of Tunis El Manar, Tunis, Tunisia; 3 Laboratory of Medical Parasitology, Biotechnology and Biomolecules, LR11-IPT-06, Pasteur Institute of Tunis, Tunis, Tunisia; 4 Department of Pathology, Charles Nicolle Hospital and Faculty of Medicine of Tunis, Tunis, Tunisia; 5 UPSP EGEAL Institut Polytechnique LaSalle Beauvais, Beauvais, France; 6 Laboratory of Immunology, Charles Nicolle Hospital, Tunis, Tunisia; University of Bergen, NORWAY

## Abstract

*Leishmania*-specific cytotoxic T cell response is part of the acquired immune response developed against the parasite and contributes to resistance to reinfection. Herein, we have used an immune-informatic approach for the identification, among *Leishmania major* potentially excreted/secreted proteins previously described, those generating peptides that could be targeted by the cytotoxic immune response. Seventy-eight nonameric peptides that are predicted to be loaded by HLA-A*0201 molecule were generated and their binding capacity to HLA-A2 was evaluated. These peptides were grouped into 20 pools and their immunogenicity was evaluated by *in vitro* stimulation of peripheral blood mononuclear cells from HLA-A2^+^-immune individuals with a history of zoonotic cutaneous leishmaniasis. Six peptides were identified according to their ability to elicit production of granzyme B. Furthermore, among these peptides 3 showed highest affinity to HLA-A*0201, one derived from an elongation factor 1-alpha and two from an unknown protein. These proteins could constitute potential vaccine candidates against leishmaniasis.

## Introduction

Leishmaniasis represents a heterogeneous group of diseases with an estimated incidence of 2 million cases annually worldwide [1]. They are caused by protozoan parasites of the genus *Leishmania* and are transmitted by the bite of infected sand flies. The disease is characterized by a spectrum of clinical manifestations determined by the species of *Leishmania* and the immune response of the host to the parasite [2]. It ranges from asymptomatic infections to cutaneous or fatal visceral forms. Most individuals who developed leishmaniasis or symptomless infection are resistant to subsequent infections, which makes vaccine development rational [3]. Studies of anti-*Leishmania* vaccine candidates have advanced in recent years due to the understanding of the cell-mediated immunological mechanisms for controlling infection. However, no efficient vaccine is available for human use as of today and *Leishmania* vaccine development has proven to be a difficult and challenging task.

In common with other intracellular pathogens, cellular immune responses are critical for protection against leishmaniasis [4]. Considerable evidence suggests that *Leishmania major* infection induces the development of a Th1 response that not only controls the primary infection but also results in a lifelong immunity to reinfection. Protection against *Leishmania* infection has been shown to involve CD4^+^ and CD8^+^ T cells [5–9]. Indeed, peripheral blood mononuclear cells (PBMCs) obtained from individuals with active or healed localized cutaneous leishmaniasis proliferate and produce Th1 type cytokines, when stimulated *in vitro* with *Leishmania* antigens [10–12]. However, previous reports indicate the implication of CD8^+^ T cells in immunoprotective mechanisms in CL as well as the establishment of a Th1 response, mainly through the production of IFN-γ [12] Although cytokine production is thoroughly analyzed, the involvement of cytotoxic activity in protection remains undefined.

Previously, we have shown that cytotoxic activity specific of *Leishmania major* (*L*. *major*) is developed by individuals living in areas of *L*. *major* transmission [13] and seems to play a crucial role in resistance to re-infection (Louzir H, 2005, unpublished data). Similar data suggest that CD8^+^ T cells may have a protective role in subclinical infection [14]. Contrastingly, evidence has been accumulated regarding the role of CD8^+^ T cells in the pathophysiology of CL. Indeed, these cells have been involved in the chronicity of *Leishmania* infection by exacerbating the tissue lesions, as described in mucocutaneous leishmaniasis caused by *L*. *braziliensis* [14–16]. Such controversy regarding the role of cytotoxicity in the pathogenesis of human leishmaniasis indicates that the functions of CD8^+^ T cells remain to be established. Furthermore, conflicting data about the route of activation of CD8^+^ T cells in leishmaniasis exist, since *Leishmania* resides within the parasitophorous vacuole of the macrophage and it is not clear how these cells present *Leishmania* antigens to CD8^+^ T cells through class I MHC [17–19]. Several data suggest that external or secreted *Leishmania* antigens are able to reach macrophage cytosol to be presented by class I HLA molecules, which is a prerequisite for CD8^+^ T cell activation [17–19].

Previously, we also have characterized a set of 33 *Leishmania* proteins that are potentially secreted by the parasite in the phagolysosomal vacuole [20].

Herein, we have first used immuno-informatic tools to select nonameric peptides derived from the 33 *Leishmania major* excreted/secreted (*Lm*ES) proteins previously described based on the binding motifs of the class I MHC: HLA-A*0201, which is the most frequent HLA allele in the Tunisian population (32.5%) [21]. Potentially ES proteins have been reported to contain antigens highly immunogenic and protective in vaccine models [17, 22–25]. Evidence has been shown regarding the immunogenicity of *Leishmania* ES proteins recovered from human cutaneous leishmaniasis [26]. *In silico* peptide prediction was followed by experimental validation of the capacity of these peptides to bind to HLA-A2 and the analysis of their immunogenicity in naturally-exposed individuals.

## Materials and Methods

### Selection of study subjects

Peripheral blood was obtained from 6 HLA-A*0201 positive and 6 HLA-A*0201 negative donors recovered from zoonotic cutaneous leishmaniasis (ZCL) living in an area of high transmission of *L*. *major* parasite (Central Tunisia). These individuals were selected based on (i) clinical criteria showing the presence of ZCL scars, (ii) positivity of the leishmanin skin test (LST) reactivity, and/or (iii) positive lymphoproliferative response to soluble *Leishmania* antigens (SLA) [immune individuals]. Screening of HLA-A*0201 positive individuals was done using a lymphocytotoxicity test. HLA subtype A*0201 was confirmed by PCR using HLA SSP ABC Typing Kit (One Lambda Inc., Canoga Park, CA). HLA-A*0201 positive healthy individuals living outside endemic areas without any lymphoproliferative response to SLA were included as control groups. The main clinical and laboratory features of the selected individuals are described in Table 1. This study has obtained the Ethical Committee approval of the Pasteur Institute of Tunis (protocol number 07–0018). Individuals were included in the study after providing informed written consent.

**Table 1:** Clinical and laboratory main features of the study subjects.

	Sex	Age(years)	LCZ scars(Y/N)	LST (mm)	Proliferaion (SLA) SI	HLA-A Typing	
ZCL04	F	58	Y	8,5	41.87	A2/01	A24
ZCL05	F	50	Y	7	63.22	A2/01	A24
ZCL23	F	40	Y	ND	20.42	A2/01	A1
ZCL25	M	41	N	7	44.62	A2/01	A26
ZCL29	M	32	N	10	45.4	A2/01	A30
ZCL34	M	46	N	ND	35.68	A2/01	A30
ZCL01	M	39	Y	12	109	A1	A28
ZCL03	F	42	Y	7.5	79	A11	A32
ZCL07	F	41	Y	8.5	14	A26	A30
ZCL14	F	24	Y	14	19	A1	A23
ZCL22	F	20	N	ND	12.5	A24	A11
ZCL24	F	24	Y	ND	6.8	A3	A26
T1	F	50	N	ND	1.6	A2/01	A25/01
T2	F	42	N	ND	2.02	A2/01	A30/01

ND: Not determined; SI: Stimulation Index; SLA: Soluble *Leishmania* Antigens

LST: Leishmanin Skin Test; F: Female; M: Male; Y/N: Yes/No.

### Epitope prediction and peptide synthesis

A set of 33 *L*. *major* genes encoding proteins that are potentially ES proteins by the parasite have previously been described in our laboratory [20].

All protein sequences were submitted to analysis by computerized HLA-binding prediction based on the freely accessible online databases: “Syfpeithi”: http://www.syfpeithi.de/bin/MHCServer.dll/EpitopePrediction.htm, HLA-peptide binding prediction site supplied by: “BIMAS”: http://www-bimas.cit.nih.gov/molbio/hla_bind, “RANKPEP”: http://www.http://bio.dfci.harvard.edu/MIF/RANKPEP, and “NetMHC”: http://www.cbs.dtu.dk/services/NetMHC. “Syfpeithi”, “BIMAS”, and “NetMHC” programs provide peptide sequences that are likely to be presented by the HLA-A*0201 molecules.

The probability for the peptides to be cleaved in the proteasome was predicted by “RANKPEP” along with a ranking or score. All peptides predicted with at least 3 softwares were selected and purchased from Intavis Bioanalytical Instruments (Cologne, Germany). Stock solutions of single peptides (20mg/mL) were produced by dissolving freeze-dried peptides in DMSO (Sigma-Aldrich, St. Louis, MO) and kept at -80°C until use.

### Parasites

*L*. *major* (Zymodeme MON25; MHOM/TN/94/GLC94) isolated from skin lesions of patients with CL was used in the present study. Parasites were cultivated on NNN medium at 26°C and then were progressively adapted to RPMI 1640 medium (Sigma-Aldrich, St. Louis, MO) containing 2mM L-Glutamine (Sigma-Aldrich, St. Louis, MO), 100U/mL Penicillin (Sigma-Aldrich, St. Louis, MO), 100mg/mL Streptomycin (Sigma-Aldrich, St. Louis, MO), and 10% heat-inactivated fetal calf serum (Sigma-Aldrich, St. Louis, MO). Stationary-phase metacyclic promastigotes were used to infect macrophages.

### Cell line

The T2 cell line is a human tumor cell line that expresses HLA-A*0201 and lacks TAP1 and TAP2 transporters [T2 (174 x CEM.T2), (ATCC^®^ CRL-1992^TM^)]. It was kindly provided to us by Dr. Salem Chouaib (Gustave Roussy Institute, France).

### Detection of peptides binding to HLA-A*0201 molecules on T2 cells

The affinity of peptides for HLA-A*0201 molecules was evaluated by using the stabilization assay as previously described [27]. Briefly, T2 cells were incubated with human β2-microglobulin at a final concentration of 10μg/mL in the presence or not of peptides at 10μg/mL for 16h at 37°C in 5% CO_2_. Cells were then incubated with 5μg/mL Brefeldin A (Sigma-Aldrich, St. Louis, MO) for 2h at 37°C. Expression of HLA-A*0201 on T2 cells was then determined by staining with fluorescein isothiocyanate-labelled anti-HLA-A2 antibody (BD Biosciences, San Jose, CA) and analyzed by flow cytometry using FACScan (BD Biosciences, San Jose, CA). Results were expressed in relative fluorescence intensity (RFI) calculated as the percentage increase of the mean fluorescence above that of the negative controls [28].

### *In vitro* stimulation of PBMCs with peptides

To assess whether the selected peptides could stimulate or not CD8^+^ T cells, we have analysed the induction of GrB and IFN-γ by stimulated PBMCs from healed ZCL individuals. PBMCs separated from heparinized blood samples using Ficoll-Hypaque (Sigma-Aldrich, St. Louis, MO) density gradient centrifugation were resuspended in RPMI 1640 medium (Sigma-Aldrich, St. Louis, MO) supplemented with 2mM L-Glutamine (Sigma-Aldrich, St. Louis, MO), 1mM sodium pyruvate (Gibco, Invitrogen, Grand Island, NY), 100U/mL Penicillin (Sigma-Aldrich, St. Louis, MO), 100μg/mL Streptomycin (Sigma-Aldrich, St. Louis, MO), 10mM HEPES (Gibco, Invitrogen, Grand Island, NY), 20μg/mL Gentamicin (Gibco, Invitrogen, Grand Island, NY), 1X non-essential amino acids (Gibco, Invitrogen, Grand Island, NY), 2-mercaptoethanol (Gibco, Invitrogen, Grand Island, NY), and 10% (v/v) heat-inactivated human AB serum (Sigma-Aldrich, St. Louis, MO), [complete medium] at a concentration of 1.0x10^6^ cells/mL. Peptide pools were prepared instantly by dilution with phosphate buffered saline and then added to the cell culture at a final concentration of 1μg/mL. In some experiments, peptides were added separately to the culture at a concentration of 20μg/mL. As positive control, PBMCs were stimulated with 10ng/mL of phorbol 12-myristate 13-acetate (PMA) (Sigma-Aldrich, St. Louis, MO) and 50ng/mL Ionomycin (Sigma-Aldrich, St. Louis, MO). All cultures were incubated at 37°C in 5% CO_2_ for 5 days. Culture supernatants were then harvested and frozen at -80°C until use.

### Granzyme B, IFN-γ, and IL-10 ELISA assays

Granzyme B (GrB), IFN-γ, and IL-10 levels in culture supernatants were quantified with an enzyme-linked immunosorbent (ELISA) assay (MABTECH AB, Nacka Strand, Sweden) for the first one and OptEIA set ELISA assay kit (BD Biosciences, San Jose, CA) for the others. The results were expressed as pg/mL based on the standards provided by the kits. Quantification thresholds were fixed to 100pg/mL for GrB, 45pg/mL for IFN-γ, and 20pg/mL for IL-10.

### Statistical analyses

Statistical analyses were carried out by using GraphPad Prism 5.0 (GraphPad Software, San Diego, CA). Mann-Whitney test was used to compare the induction of GrB and IFN-γ between the different study groups. Correlation between GrB and IFN-γ levels induced by peptide pools or individual peptides were estimated by use of Spearman’s rank order correlation coefficient. A classification of the peptide pools according to their induction of GrB was achieved using Matlab 7.0 (Mathworks, Inc., Natick, MA). A Kruskal-Wallis test was performed to compare the rank of peptide pools.

## Results

### Selection of potential HLA-A*0201-binding peptides within *Lm*ES proteins

**Fig 1:**
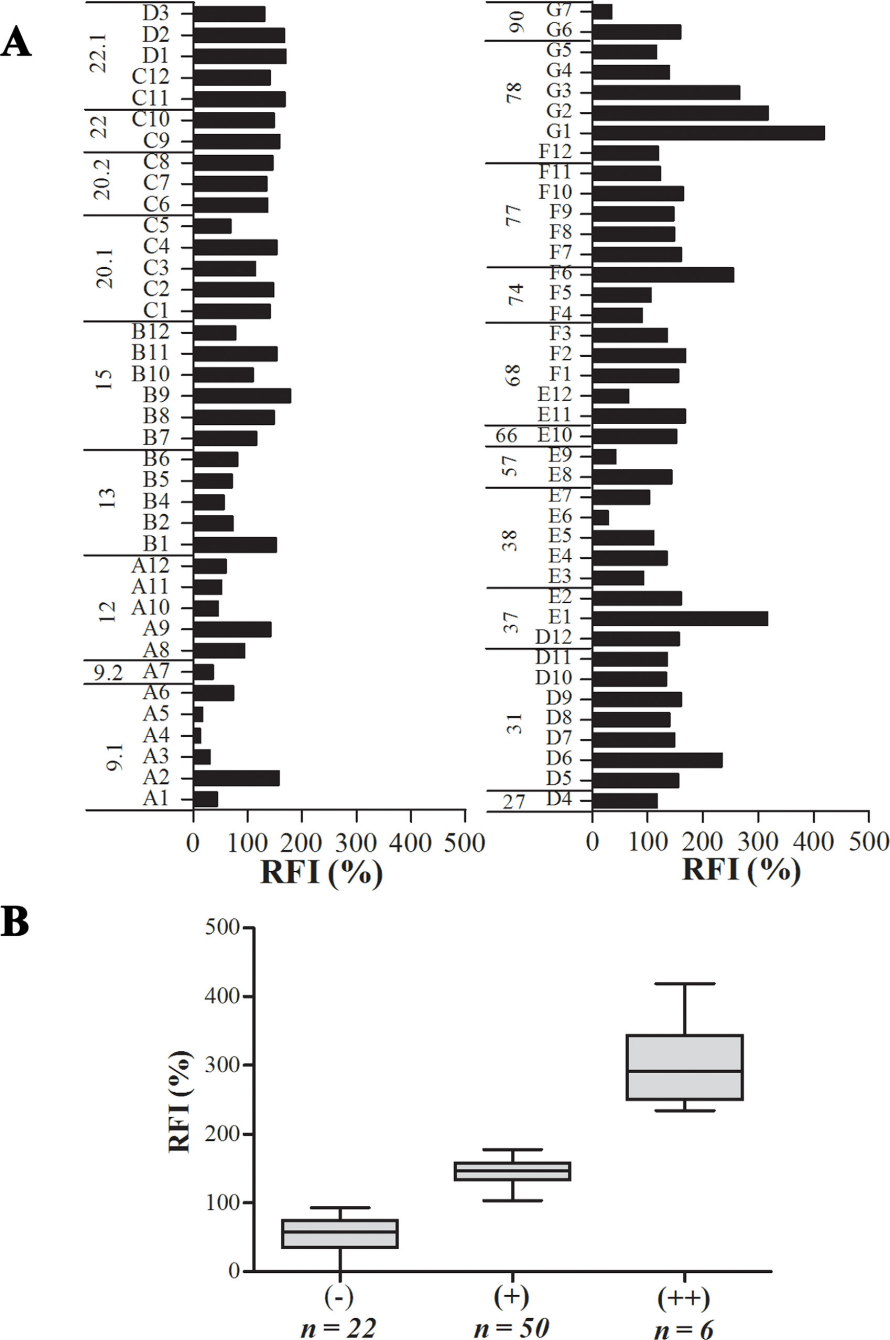
Binding affinity of *Lm*ES peptides to HLA-A*0201 molecules. The affinities of selected peptides were determined by class I HLA stabilization assay. (A) Results for individual peptides. T2 cells were initially incubated with 100μg (final concentration) of each of the peptides/mL for 16h at 37°C, followed by incubation at 37°C for 2h in presence of Brefeldine A. HLA-A2 expression on these cells was analyzed by flow cytometry using the BB7.2 antibody. MHC stabilization efficiency for each peptide was calculated as the percentage increase of the mean fluorescence above that of the negative controls. Results were expressed as relative fluorescence intensity (RFI). (B) Box plot results. ++: RFI ranges from 200 to 300%, +: RFI ranges from 100 to 200%, and -: RFI ranges from 0 to 100%.

The sequence of 33 different clones of potentially ES proteins has been used. Based on computer software predictions, putative class I HLA-restricted T cell epitopes were identified. Twenty proteins were able to generate a total of 78 nonameric peptides that could be loaded by HLA-A*0201 molecule (Table 2). Subsequently, we have evaluated the binding affinity of these peptides to HLA-A*0201 molecules by class I HLA stabilization assay. This assay measures the increase of HLA-A*0201 molecules induced on T2 cells following exposure to exogenous HLA-A*0201 binding peptides, with high affinity peptides inducing HLA-A*0201 up-regulation more strongly than low-affinity peptides. Individual results are shown on Fig 1A. The 78 tested peptides were classified into 3 groups regarding the percentage of RFI. Six peptides namely, D6, E1, F6, G1, G2, and G3 showed the highest percentage of RFI increase (RFI > 200%), 50 peptides showed intermediate affinity (RFI ranges from 100 to 200%), and 22 peptides had a weak affinity (RFI < 100%) (Fig 1B).

**Table 2:** Characteristics of *in silico* predicted HLA-A*0201-restricted peptides for *Lm*ES proteins.

Protein	peptide	Start Position	SEQUENCE	RANKPEP^a^	SYFPEITY^b^	BIMAS^a^	NET MHC MATRIX^a^	NET MHC ANN^a^
	**A1**	85	ALQEETHVL	82	75%	35.9102	25.522	101
	**A2**	427	YMAQKAEEV	74.05	69.44%	113.2229	20.067	48
**Pr 9.1 (LmjF.14.0820)**	**A3**	259	KLTVSSAAV	93	-	-	23.091	1580
	**A4**	92	VLGSHVQTL	86	75%	83.5270	25.372	-
	**A5**	182	LLRQETARL	82	72.22%	-	23.016	1468
	**A6**	362	HLMGQLNEL	79	83.33%	-	25.566	274
**Pr 9.2(Ribosomalprotein S18)**	**A7**	107	RLRDDLERL	70	69.44%	7.5019	20.177	1437
	**A8**	45	YLLDVSTLL	94	6.44%	1490.7110	26.521	100
	**A9**	198	NLIDFNFKL	72	72.22%	1930.3919	20.772	152
**Pr 12 (Ubiquitin**	**A10**	185	LLKDSFAFL	85	66.66%	-	22.603	653
**protein ligase: LmjF.07.0280)**	**A11**	230	CLLDSFKEL	75	66.66%	615.7285	22.474	-
	**A12**	167	VLEENRTTL	73	66.66%	-	20.196	-
	**B1**	9	VLCALLFCV	68	72.22%	1577.3003	25.092	562
	**B2**	387	KLHPVYDKV	66	69.44%	178.9225	24.676	318
**Pr 13 (LmPDI)**	**B4**	259	ALKGSLVAV	91	83.33%	-	22.517	344
	**B5**	157	EMASMITKV	88	63.88%	-	-	1171
	**B6**	63	DMLAGIATL	69	80.55%	-	-	716
	**B7**	321	LLSAQIARL	93	77.77%	83.5270	23.94	771
	**B8**	256	LLFDELTAL	84	80.55%	1267.1043	24.159	174
**Pr 15 (LmjF.15.0410)**	**B9**	737	RLMQCVQQL	81	69.44%	181.7940	23.118	1445
	**B10**	33	SLVVVSASL	88	72.22%	-	23.241	5167
	**B11**	29	SLCRSLVVV	86	77.77%	-	23.281	5111
	**B12**	22	HLVAPLASL	82	80.55%	-	23.516	2134
	**C1**	395	ALNDALWAV	96	80.55%	4919.0652	27.307	45
	**C2**	77	RLLVDLAQL	83	77.08%	181.7940	21.354	-
**Pr 20.1 (Chaperonin**	**C3**	175	IVVDAIMSV	101	-	97.5615	-	465
**subunit alpha: LmjF.32.3270)**	**C4**	367	VIAGTSNAV	77	69.44%	-	-	835
	**C5**	128	AMREALRYL	76	72.22%	-	-	349
	**C6**	213	GVFDAAISI	82	-	13.8482	-	600
**Pr 20.2 (LmjF.36.2650)**	**C7**	69	QVGAFLEGL	50	55.55%	8.0051	-	-
	**C8**	145	GLDYSEELL	46	55.55%	4.1870	-	-
**Pr 22 (LmjF.05.0710)**	**C9**	111	RVAASVAAV	95	63.88%	13.9973	-	10621
	**C10**	221	GTDDTVAAV	64	63.88%	3.6438	-	8253
	**C11**	141	TIPSFIVRV	88	69.44%	83.5841	-	2164
	**C12**	68	RLLEGSAIM	79	61.11%	30.8995	-	582
**Pr 22.1 (Ribosomal**	**D1**	127	LIQQRHIAV	66.05	58.33%	16.2578	-	1987
**protein S9: LmjF.36.1250)**	**D2**	134	AVAKQIVTI	95	66.66%	-	-	8403
	**D3**	103	ILERRLQTI	78	69.44%	-	-	2278
**Pr 27 (similar to LAEL147_000045800)**	**D4**	24	NMMAVVGLL	81	63.88%	17.0684	-	6564
	**D5**	836	KLEDEVFAL	83	72.22%	261.7205	23.158	170
	**D6**	892	ELLGNLEEV	79	75%	21.7519	24.38	535
**Pr 31 (LmjF.34.0680)**	**D7**	690	RMADEVQRL	77	69.44%	145.4898	20.464	520
	**D8**	135	RLAVSLHEL	81	80.55%	49.1335	22.162	-
	**D9**	648	LLGPAYQSI	78	-	26.6036	-	729
	**D10**	781	VIAEEPLYV	77	-	366.6129	-	1130
	**D11**	40	PLSAVISPV	81	-	-	25.673	641
	**D12**	320	LLPAPLVSV	90	86.11%	271.9483	26.986	504
**Pr 37 (LmjF.36.3860)**	**E1**	575	MLLWTAVAV	82	69.44%	437.4821	25.381	162
	**E2**	135	YLRTFPAAL	70	72.22%	-	27.977	-
	**E3**	336	RLAGFLAGL	78	88.88%	186.7074	24.784	516
	**E4**	385	CLALIAWRV	67	61.11%	521.1640	21.709	605
**Pr 38 (similar to LTRL590_180019400)**	**E5**	69	VVAGMLRWV	65	63.88%	26.1750	-	1295
	**E6**	134	PLSPATRRL	64	58.33%	-	22.415	1722
	**E7**	92	MVLNAMAWL	78	-	148.7302	-	2967
**Pr 57 (Ribosomal**	**E8**	53	KIMEAITVV	105	72.22%	478.8259	-	384
**protein S16: LmjF.26.0880/ LmjF.26.0890)**	**E9**	115	FLAYDKFLL	115	61.11%	569.9488	-	343
**Pr 66 (LmjF.26.0880/ LmjF.26.0890)**	**E10**	461	HIFDRVAGV	78	75%	-	-	361
	**E11**	75	ALNQFTKVL	79	69.44%	33.2826	22.002	2323
**Pr 68 (Ribosomal**	**E12**	179	AIVKDMARL	88	61.11%	6.7559	-	7557
**protein L7/**	**F1**	136	GLQEVTRAI	83	66.66%	8.5549	-	717
**L12-like protein: LmjF.07.0500)**	**F2**	153	VIANNVDPV	69	69.44%	18.3225	-	2773
	**F3**	211	TLKNLIRSV	87	72.22%	-	-	735
**Pr 74 (elongation**	**F4**	215	TLLDALGML	93	75%	96.8962	21.586	1262
**factor proteasome**	**F5**	137	ALLAFTLGV	90	80.55%	977.9011	27.727	287
**1-alpha: LmjF.17.0082)**	**F6**	142	TLGVKQMVV	78	58.33%	28.5163	-	2243
	**F7**	263	KLVRELFRV	90	69.44%	3247.3829	25.63	506
**Pr 77 (Probable**	**F8**	306	TMLELLTQL	89	75%	538.3123	20.999	342
**regulatory ATPase**	**F9**	404	ALRERRMKV	80	72.22%	21.6724	20.701	677
**(*L*. *major*): LmjF.13.1090)**	**F10**	149	LLHDRQHSI	73	66.66%	72.7166	-	87
	**F11**	187	GLEQQIQEI	81	66.66%	-	-	623
	**F12**	150	MLQTNSLAL	85	61.11%	36.3161	24.802	1677
	**G1**	**24**	SLQFSAFLL	68	58.33%	123.9019	21.087	2809
	**G2**	**117**	FMEVFGMLV	50	52.77%	56.1955	-	148
**Pr 78**	**G3**	**83**	MLVQSCTSI	90	55.55%	-	-	1781
	**G4**	110	VVSVLTHSV	69	58.33%	-	-	2390
	**G5**	106	WIPPVVSVL	56	66.66%	-	-	5645
**Pr 90 (Ribosomal**	**G6**	286	KIYQIGRSV	61	61.11%	21.4220	-	352
**protein L3: LmjF.32.3130)**	**G7**	282	QLNKKIYQI	88	69.44%	23.9954	-	-

^a^: Results expressed as score.

^b^: Results expressed as percentage calculated according to the highest score (= 36).

### Stimulation with peptide pools induces production of GrB

Given their large number and to test their immunogenicity *in vitro*, the predicted peptides were compiled into 20 pools as shown in Table 3. Each pool contains peptides belonging to the same protein. Pools were tested for their ability to induce GrB secretion by PBMCs obtained from 5 HLA-A*0201^+^-immune donors and 2 HLA-A*0201^+^ healthy donors. Surprisingly, low IFN-γ levels, not exceeding 45pg/mL (quantification threshold), were detected in culture supernatants of PBMCs obtained from immune individuals, stimulated with the different peptide pools. Similar results were obtained for IL-10, which was detected at low levels (ranging from 20 to 120pg/mL) in only one immune individual. Stimulation of PBMCs from these individuals with SLA or PMA/Ionomycin showed high levels of IFN-γ (*data not shown*).

**Table 3:** Setup of peptide pools.

Protein	9.1	9.2	12	13	15	20.1	20.2	22	22.1	27	31	37	38	57	66	68	74	77	78	90
Pool*	1	2	3	4	5	6	7	8	9	10	11	12	13	14	15	16	17	18	19	20
	A1	A7	A8	B1	B7	C1	C6	C9	C11	D4	D5	D12	E3	E8	E10	E11	F4	F7	F12	G6
	A2		A9	B2	B8	C2	C7	C10	C12		D6	E1	E4	E9		E12	F5	F8	G1	G7
	A3		A10	B4	B9	C3	C8		D1		D7	E2	E5			F1	F6	F9	G2	
	A4		A11	B5	B10	C4			D2		D8		E6			F2		F10	G3	
	A5		A12	B6	B11	C5			D3		D9		E7			F3		F11	G4	
	A6				B12						D10								G5	
											D11									
***n***	6	1	5	5	6	5	3	2	5	1	7	3	5	2	1	5	3	5	6	2

* Numbers 1 to 20 refer to peptide pools.

*n*: Number of peptides made up for each protein.

**Fig 2:**
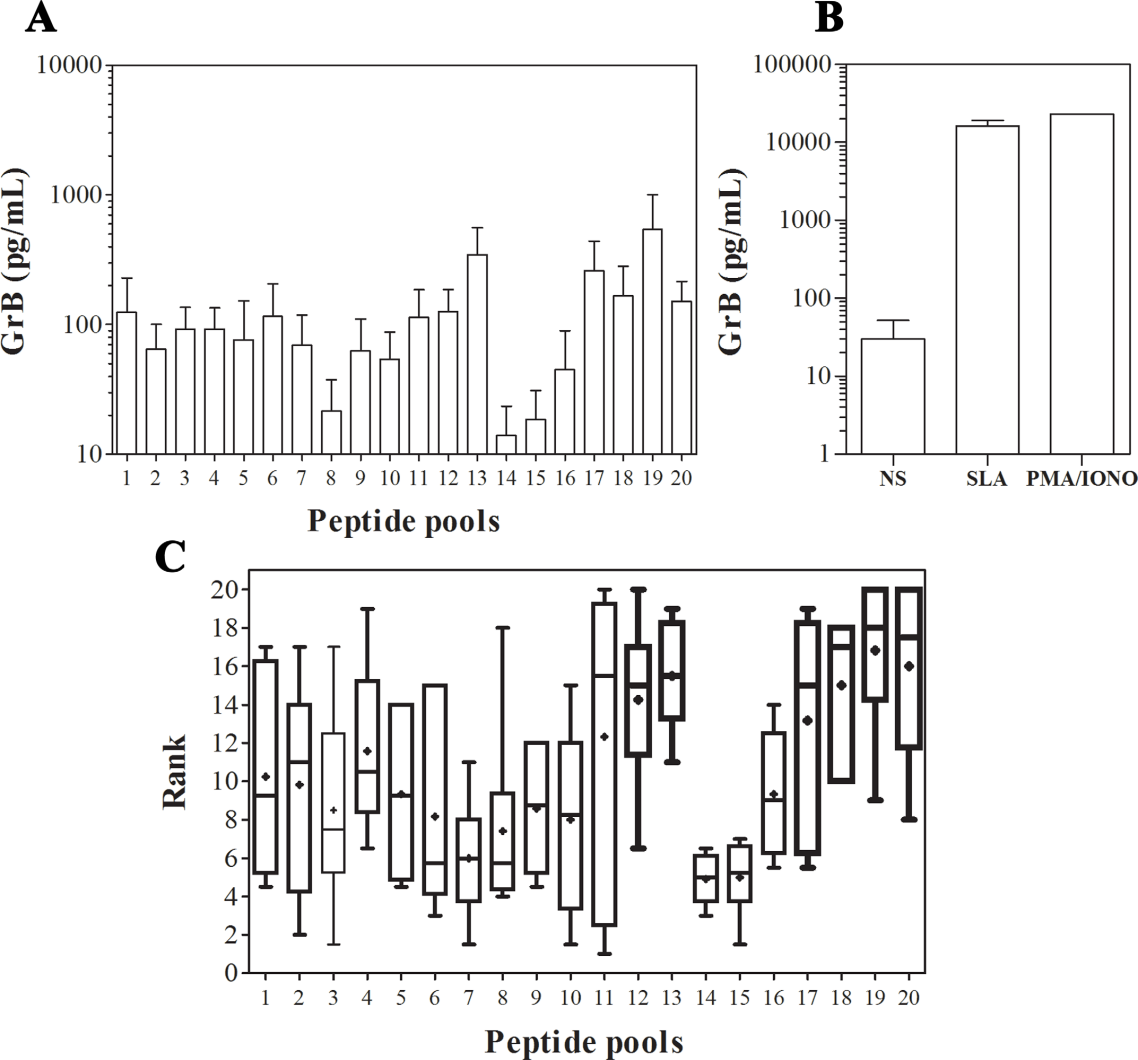
Peptides grouped in pools induced GrB production. (A) PBMCs from 5 HLA-A*0201^+^-donors with a history of ZCL in response to stimulation with peptide pools at a final concentration of 1μg/mL per peptide or (B) SLA (10μg/mL) and PMA/Ionomycin (10ng/mL and 50ng/mL, respectively) as positive controls. GrB production was assessed in culture supernatants using ELISA. (C) Rank of the peptide pools. **+**: mean pool rank, **-**: median.

As shown in Fig 2A, peptide pools induce variable levels of GrB in culture supernatants of PBMCs obtained from immune individuals. In contrast, for healthy donors no GrB production could be induced. Considering the variability of detected GrB levels, we have resorted to a ranking method (Fig 2C). Classification of the 20 peptide pools corresponding to the 20 different *Lm*ES proteins was done according to their capacity to induce GrB production. The concept consists of computing the rank of the different pools for each individual and then calculating the mean rank of each pool for the 5 individuals tested. Interestingly, the Kruskal-Wallis test has revealed that the highest GrB levels were induced by the peptide pools P19, P20, P13, P18, P12, and P17 corresponding respectively to the proteins Pr78, Pr90, Pr38, Pr77, Pr37, and Pr74. GrB levels measured in culture supernatants of PBMCs stimulated with these peptide pools were significantly higher compared to those induced by the other ones (*p* = 0.0002). Taken together, these results allow us to rank 6 proteins among the potentially ES proteins as best generators of peptides that are recognized by PBMCs of HLA-A*0201^+^-immune individuals.

### Evaluation of GrB and IFN-γ production by PBMCs stimulated with individual peptides

**Fig 3:**
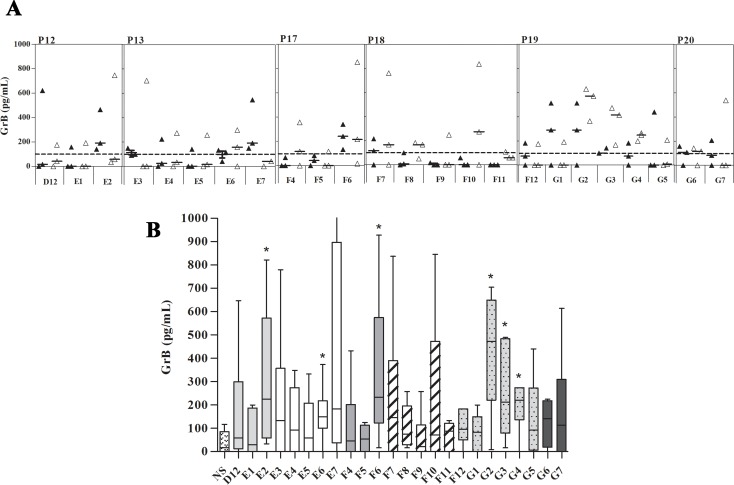
GrB induction by the selected peptides. (A) PBMCs from HLA-A*0201^+^ (black mark) and HLA-A*0201^-^ (white mark) -healed ZCL individuals were stimulated with selected peptides separately. The supernatant was collected after 5 days of incubation and assayed for GrB production. (B) Peptides E2, E6, F6, G2, G3, and G4 induced the highest levels of GrB.

All peptides belonging to the selected proteins were tested separately for their capacity to induce GrB and IFN-γ. PBMCs obtained from 3 HLA-A*0201^+^ and 3 HLA-A*0201^—^immune donors were stimulated with the different individual peptides, then GrB and IFN-γ levels were measured in culture supernatants. We have used PBMCs obtained from 2 HLA-A*0201^+^-healthy individuals as negative controls. It should first be mentioned that levels of IFN-γ detected in culture supernatants were very weak (< 45pg/mL) in the individuals tested with most of the peptides. Regarding GrB, no production could be detected in supernatants of PBMCs obtained from negative controls in response to stimulation with any of these peptides (*data not shown*). Interestingly, variable levels of GrB were detected in HLA-A*0201^+^ and HLA-A*0201^—^immune donors (Fig 3A). However, there was no statistically significant difference between HLA-A*0201^+^ and HLA-A*0201^—^immune individuals (p > 0.05 for all tested peptides). Taken together, 6 peptides (E2, E6, F6, G2, G3, and G4) among the 24 tested have been shown to induce the highest levels of GrB (Fig 3B). Furthermore, 5 peptides out of the 6 selected ones stabilized HLA-A2 molecule on T2 cells with high (F6, G2, and G3) or intermediate (E2 and G4) affinity. Only E6 showed no affinity for HLA-A2 molecule.

## Discussion

For a long time, it has been a consensus that a Th1 dominant response promotes IFN-γ production, induces lesion healing, and controls parasite burden [7]. Based on this, different vaccine candidates have been selected. CD8^+^ T cells play a major role in controlling leishmaniasis, since growing evidence did prove their participation in the immune response against different *Leishmania* species studied in experimental models and humans [29, 30]. Few studies have focused on the identification of *Leishmania* epitopes that can be presented by class I MHC molecules to CD8^+^ T cells [31, 32]. Currently, there are no well-defined *Leishmania* CD8^+^ T cell epitopes, which has made it difficult to investigate how CD8^+^ T cell activation occurs in leishmaniasis. Antigen-presenting cells, such as macrophages and dendritic cells have been shown to be able to capture, process, and present in a class I MHC-restricted manner various exogenous antigens including those derived from intracellular pathogens like *Leishmania* parasites [17, 33].

Previously, we have characterized 33 *Leishmania* genes coding for proteins that are probably released by the parasite in the phagolysosomal vacuole [20].

Herein, we have analyzed these potentially *Lm*ES proteins in an attempt to identify HLA-A*0201-binding peptides able to activate CD8^+^ T cells. We have identified 6 epitopes: E2, E6, F6, G2, G3, and G4 that are able to induce GrB production by PBMCs obtained from immune individuals. These peptides derived from the sequence of the Pr37, Pr38, Pr78, and Pr74 proteins. Our study is not exhaustive since the choice of the 33 potentially ES protein sequences was made out of more than 8,000 parasite protein-coding genes. In fact, there are probably additional *Leishmania* ES proteins that have not been described as of yet. Moreover, it is quite possible that non-excreted parasitic antigens able to generate CD8^+^ T cell epitopes do also exist.

Our hypothesis is clear and our approach is simple. We have assumed that *Lm*ES proteins may generate peptides that could be presented to CD8^+^ T cells. This approach oriented us towards 4 proteins of interest. Pr74 corresponding to elongation factor-1 alpha (EF-1α), which is a multifunctional protein essentially involved in protein biosynthesis and parasite survival in infected macrophages [34–39]. Indeed, the presence of *Leishmania* EF-1α in the cytosol of infected macrophages has also been demonstrated [37, 38]. Interestingly, this protein was one of the leishmanial antigens that was used for construction of the vaccine LeishDNAvax composed of MIDGE-TH1 vectors encoding 5 conserved leishmanial antigens: KMP11, TSA, CPA, CPB, and Pr74 [40]. The 3 remaining proteins were described as potentially secreted by the parasite but do not correspond to any proteins described in sequence libraries. Among them, 2 proteins Pr37 and Pr78 contain signal peptide sequences and one, Pr38 was predicted to be secreted via non-classical pathways [20].

In this study, the selection of peptides was performed using the computer-based prediction method, which constitutes a useful tool for peptide identification. However, this method is unable to predict all peptide sequences. Thus, some interesting and immunogenic peptides could be missed out and prevented from being tested in the immune response, only because *in silico* methods could not predict them. So the best way is to use overlapping peptides to scan all protein sequences as performed by Basu and collaborators regarding peptide identification belonging to the protein Kmp11 [31]. Nonetheless, this method could not be applied in our study because it would have given us thousands of peptides to test, which was currently not feasible for all proteins tested here. Moreover, Pelte and collaborators have previously identified one single stimulating peptide, which did not stabilize HLA-A*0201 expression on T2 cells and could therefore not be presented by HLA-A*0201 [41]. The paradox of T cell recognition of a peptide that fails to bind to HLA-A2 could be explained by the fact that peptides could be recognized after binding to other class I HLA molecules carried out by the patients, which could subsequently present the epitope to specific T cells.

The next step was to analyse the immunogenicity of the antigenic peptides in naturally-infected individuals. In addition to their capacity to bind to class I MHC molecules, we have assumed that these peptides exist in large quantities in the intracellular phagolysosomal vesicle. Consequently, in natural infection some peptides predicted to have high affinity in theoretical and functional tests could fail to induce significant immune response since they are not secreted or because they are in different cellular structures. By contrast, some low-affinity peptides can still be presented by class I MHC molecules because of their abundance in the intracellular phagolysosomal vesicle. For these reasons, all predicted peptides were compiled in pools and their immunogenicity tested in HLA-A*0201^+^-ZCL recovered individuals. Pooling peptides has been used in many previous studies [28, 32] and does not seem to be a limiting factor [42, 43] considering that all peptides are predicted with almost equal affinity for HLA binding and with same stimulatory concentrations in cultures. Unexpectedly, weak levels of IFN-γ were detected in culture supernatants of PBMCs stimulated with the different peptide pools. This cannot be attributed to the inhibition of IFN-γ production by IL-10, which was not detected in these culture supernatants. This could rather be explained by the possibility of low frequency of memory CD8^+^ T cells due to stimulation conditions. In fact, in the present study PBMCs were stimulated with peptide pools without adding IL-2 or anti-CD48 as done by Seyed and collaborators [32]. Our results are similar to those described in other studies using different read out systems for the IFN-γ detection in T cells, such as flow cytometry [41] or ELISPOT [28]. Results of these two studies showed a weak production of IFN-γ induced by only few peptides among those selected by using bioinformatics.

Further, we have shown here that variable levels of GrB were induced by the different peptide pools, which led us to rely on the ranking method. Thus, we have selected 6 proteins as the best generators of peptides recognized by PBMCs obtained from HLA-A*0201-immune individuals. Consequently, we have analyzed separately the immunogenicity of all peptides belonging to these proteins. The highest GrB levels were detected in supernatants of PBMCs stimulated with the peptides E2, E6, F6, G2, G3, and G4. Unexpectedly, these peptides have also induced GrB production in HLA-A*0201-negative immune individuals. Similar results have been reported by Seyed and collaborators [32]. As discussed by the authors, this could be explained by specificity overlap between supertypes of HLA molecules and would need to be further confirmed in a larger population of individuals bearing other HLA-A alleles [32, 44]. To achieve that, we will be extending our study to map potential CD8^+^ T cell epitopes restricted to other common class I HLA alleles.

To better trigger the specific response to our peptides, several experiments are planned, i.e., establishing “short-term” cell lines specific of the selected peptides and analyzing their ability to induce the production of GrB, IFN-γ, IL-2, and IL-10 when co-cultured in the presence of the T2 cell line pulsed with each of the peptides, and used as antigen-presenting cells.

In conclusion, we have identified novel HLA-A*0201-restricted immunogenic CD8^+^ T cell epitopes derived from potentially *Lm*ES proteins using *in silico* prediction and functional studies on PBMCs obtained from immune individuals. Proteins we have identified here could constitute potential candidate vaccine antigens.
